# Prevalence, Prevention and Treatment of Saddle Sores among Female Competitive Cyclists: A Scoping Review Protocol

**DOI:** 10.3390/mps3010004

**Published:** 2020-01-06

**Authors:** Keira Bury, Justine E. Leavy, Amanda O’Connor, Jonine Jancey

**Affiliations:** 1 School of Public Health, Curtin University, GPO Box U1987, Perth, WA 6845, Australia; 2 National Cycling Centre WA, PO Box 304 Mundaring, Perth, WA 6073, Australia; amandaoc@iinet.net.au

**Keywords:** saddle sores, competitive cycling, sports medicine, athletic injuries, bicycling, female athletes, folliculitis, skin diseases

## Abstract

Female cyclists are prone to a variety of injuries and illnesses that occur as a result of prolonged contact with a bicycle saddle. Saddle sores are a range of skin ailments on the buttocks, genitals and inner thigh that result from a combination of friction, heat, pressure, moisture and bacteria in the saddle area. Whilst saddle sores are reportedly common, for some cyclists, the condition may cause only mild discomfort. However, for female competitive cyclists, the condition can be an ongoing source of pain and illness affecting participation and performance in the sport. Despite many online sources for health information and products for saddle sores, it is unknown what empirical evidence exists for the prevalence and severity of saddle sores, and for the effectiveness of prevention and treatment methods. This paper outlines the protocol for a scoping review, which aims to describe the empirical evidence for the prevalence, prevention and treatment of saddle sores among female competitive cyclists. Ethics approval has been obtained for this study from Curtin University’s Human Research Ethics Committee no: HRE2019-0120. The findings from this study will contribute to the literature for injury in female sport.

## 1. Introduction

Female cyclists are known to experience a variety of injuries and illnesses that occur as a result of prolonged contact with a bicycle saddle [1,2,3]. Saddle sores are one of these conditions and are described as a range of skin ailments on the buttocks, genitals and inner thigh [4]. Saddle sores are reportedly common among both male and female, novice and competitive cyclists and result from a unique combination of physiological and mechanical conditions which create friction, heat, pressure, moisture and bacteria in the saddle area [2]. For some cyclists, saddle sores may cause only mild discomfort, however for others, including female competitive cyclists, the condition can be an ongoing source of pain and illness affecting participation and performance in the sport [5].

Saddle sores often begin as minor chafing, or as folliculitis, an inflammation of the hair follicle, and can progress into severe skin abrasion, deep skin infection and abscess [2]. Once established, saddles sores can be challenging to treat, and clinicians who are unfamiliar with the condition may be further limited by a lack of empirical evidence [6]. Female competitive cyclists are susceptible to a variety of other saddle related conditions including genital trauma, sexual dysfunction, vulvar disease and genitourinary infection [2,7,8]. To date, it appears the clinical research is dominated by these conditions, which may be perceived as more serious than saddle sores.

Health information is now widely disseminated online, and anecdotally saddle sores appear to be well documented across mainstream cycling websites and forums [9]. This may be beneficial for young women who generally find the internet an acceptable source of health information and may be less willing to discuss ‘embarrassing’ issues with training staff and physicians [9]. However, the authenticity of the information presented online is largely unknown and is often linked to the promotion of commercial products. Across these websites, there are several frequently mentioned factors for preventing saddle sores including the saddle design; the cyclist’s position on the bike; the style and quality of bike shorts (chamois); application of a lubricant barrier between the skin and bike shorts (chamois cream); personal hygiene and hair removal [10,11]. Other factors less commonly mentioned include hormone levels, nutrition and the cyclist’s training regime [11,12]. Treatment methods are also commonly cited, including rest (time off the bike), the application of heat or cold, topical applications with steroidal, anti-fungal and anti-bacterial ingredients, clothing in loose, breathable fibres and oral antibiotics [4,10,11,12,13]. The breadth of this information demonstrates the potential difficulties for female participating in competitive cycling to manage the prevention and treatment of saddle sores, particularly during periods of intense training.

Despite a plethora of online health information on saddle sores, there is little known regarding the prevalence and severity of saddle sores among female competitive cyclists, or the effectiveness of popularised methods for prevention and treatment. An understanding of the empirical evidence may assist physicians, allied health practitioners and coaches to better engage with female cyclists. This paper outlines the protocol for a scoping review, which aims to describe the evidence for the prevalence, prevention and treatment of saddle sores among female competitive cyclists.

## 2. Experimental Design

### 2.1. Study Design

A scoping review is an exploratory study that brings together evidence from disparate sources regardless of the quality. Scoping reviews do not seek to analyse the evidence, but aim to map the types of evidence, the key concepts and the gaps in research within a defined field [14]. The methodology for this scoping review is guided by the Joanna Briggs Institute Reviewers Manual 2015 Methodology for Scoping Reviews [15], which is derived from the framework developed by Arksey and O’Malley [14] and advanced by LevacColquhoun and O’Brien [16].

A preliminary search for existing reviews of saddle sores among female cyclists was conducted during November 2018. The databases searched were: ProQuest, CINAHL Plus with full text, PubMed, Scopus and SPORTDiscus with Full Text and Proquest Dissertations using the terms ‘*saddle sores*’ AND *cycling* AND *review*. The search revealed a narrative review of the literature for pelvic floor injuries among female cyclists conducted in 2016 [1]. This was a review of 12 studies that reported a range of saddle related injuries among female cyclists, some of which included saddle sores. The review highlighted three specific prevention and therapeutic measures: saddle design, saddle position, and skin creams. The authors found there to be a paucity of quality evidence in this area and recommended further research into the prevalence, prevention and treatment of saddle injury among female cyclists. Whilst the methodology for the narrative review sought to describe the evidence for a range of pelvic floor symptoms among female cyclists, this scoping review will systematically explore the evidence for the range of skin conditions described as saddle sores.

### 2.2. Review Title, Objective and Question

#### 2.2.1. Review Title

Saddle sores among female competitive cyclists: a scoping review. The review title was constructed using the population, concept, context (PCC) mnemonic, which defines the population (female competitive cyclists), concept (saddle sores) and context (all available evidence).

#### 2.2.2. Review Objective

The objective of the review is to map the available evidence and provide an overview of the prevalence, prevention and treatment of saddle sores among female competitive cyclists.

#### 2.2.3. Review Question

The review asks, ‘what evidence is there on the prevalence, prevention and treatment of saddle sores among female competitive cyclists?’ This is the primary question of the review and provides a clear structure for the search process and inclusion criteria.

Evidence will not be constrained by the date of publication or geographical region. Evidence will be included if the source:

- Is a primary study;
- Indicates the prevalence, prevention or treatment of saddle sores;
- Includes a female study population who undertake some level of competitive cycling (elite or novice).

Evidence will be excluded if the source:

- Is a secondary review/discussion/opinion of the literature;
- Is anecdotal evidence (no research methodology described);
- Does not include female study participants;
- Study participants are described as non-competitive, recreational cyclists; or
- Non-English language material.

## 3. Procedure

The full search strategy will comprise two components:

1. A database search for published and unpublished literature including primary studies and opinion articles; and
2. An advanced Google search for grey literature.

The initial search terms are (*cycling* OR *cyclist* OR *bicycle* OR *bike*) AND (‘saddle sore*’ OR *chafing* OR *boil* OR *vulva OR genital*). These terms are derived from an analysis of keywords from papers retrieved in the preliminary search and are congruent with the review title, objective and research question. They will be used to conduct a full search of five databases (EMBASE (Ovid), Medline (Ovid), PubMed, SCOPUS, SPORTDiscus) in addition to an advanced Google search. A search of ResearchGate ‘projects’ will also be undertaken. The search will be limited to title and abstract, and no date limits. The review search process will be iterative, allowing for the addition of key words and sources of evidence. The reference list of key papers will be used to search for additional citations not found in the first stage of the study selection. Authors of conference papers or abstracts will be contacted should the search fail to retrieve the study’s full-text version. These authors will be contacted twice within a two-week period to allow for inclusion in the review.

### 3.1. Study Selection

In the first stage of the study selection, the title and abstract (if applicable) from search results will be examined by two independent reviewers (K.B., C.L.). In the second stage, the full-text articles for studies that are deemed eligible will be extracted for screening by two reviewers (K.B., J.J.) independent of one another. The eligibility of the final selection of studies will be agreed upon by two reviewers (K.B., J.J.). During the final selection process, any differences in the decisions of the two reviewers will be discussed, and a third reviewer (J.E.L.) will be called upon to resolve any disagreements.

### 3.2. Charting the Results

The selected studies will be charted according to the source, which may include peer-reviewed literature, unpublished literature and grey literature. A charting form will be used to further map the results by the relevant characteristics: author(s); date of publication; country of origin; aims/purpose, study population and sample size; methodology, outcomes/key findings; and the relevance to the three elements from the scoping review question (prevalence, prevention, and treatment of saddle sores). Data charting will be calibrated by comparing the results of the two reviewers (K.B., J.J.). The third reviewer (J.E.L.) will be called upon to resolve any disagreements in the charting process.

### 3.3. Presentation of the Results

The review methods and results will be presented using PRISMA extension for scoping reviews (PRISMA-ScR) by [17]. This checklist extends the PRISMA statement for reporting systematic reviews by identifying 20 essential and two optional items for reporting the results of a scoping review. These include eligibility criteria, information sources, a description of the full search strategy from at least one database and a description of the methods for handling and charting data. An additional diagram will demonstrate the search process and the flow of evidence through the different stages of the study. Tabular presentation of the results will map the selected evidence according to the source, the study characteristics and the key findings. A narrative description will synthesise the results in line with the review objective and research question. The results from the studies will not be compared, but will be presented as a body of evidence for further comparison and will potentially identify research gaps.

### 3.4. Consultation Exercise

A consultation exercise will be conducted via an online survey with relevant stakeholders from the Australian cycling community (up to *n* = 30), including physiotherapists, competitive cycling coaches, gynaecologists and sports doctors. This stage aims to validate the findings of the scoping study and may also result in additional insights being included in the review and guide recommendations for future research [16,18]. A purposive/snowballing approach will be taken to select participants for the consultation exercise. The purposive sampling method involves identifying a sample of participants that have characteristics appropriate for the study. Snowballing allows the initial sample of participants to recommend other potential participants for the study until the sample size is met. This methodology enables researchers to consult with stakeholders who may be difficult to reach and who are likely to provide rich information [19]. Human ethics approval has been sought to conduct a consultation exercise for this review.

## 4. Expected Results

This scoping review seeks to increase understanding of what evidence there is, the main sources of information and the gaps in research in this area. The results may inform further research into saddle sores and the effectiveness of prevention and treatment interventions to guide evidence-based recommendations for female cyclists.
